# Prognostic molecular biomarkers in endometrial cancer: A review

**DOI:** 10.14312/2052-4994.2019-3

**Published:** 2019-12-03

**Authors:** J. Edgardo Hernández, Ailyn González-Montiel, Jesús C. Ceb Allos-Villalva, David Cantú, Salim Barquet, Anny Olivares-Mundo, Luis A. Herrera, Diddier Prada

**Affiliations:** 1 Unit of Biomedical Research, National Cancer Institute– Biomedical Research Institute, National Autonomous University of Mexico. San Fernando 22, Colonia Sección XVI, Delegatión Tlalpan, Mexico City, Mexico, 14080; 2 Department of Biomedical Informatics, Faculty of Medicine, National Autonomous University of Mexico, C.U., Av. Universidad 3000, Mexico City, Mexico, 04510

**Keywords:** molecular biomarkers, endometrial cancer, prognosis, overall survival

## Abstract

**Background:**

Endometrial cancer (EC) is the fourth most common malignancy in women worldwide and the most common gynecological cancer in developed countries. The endometrioid subtype has an excellent prognosis with conventional treatment; however, recurrence reduces overall survival.

**Objective:**

Describe the most relevant evidence regarding selected potential molecular biomarkers that may predict overall survival (OS), relapse-free survival (RFS), and cancer-specific survival (CSS) in EC.

**Methods:**

An exhaustive search was performed in PUBMED with the search terms endometrial cancer, molecular biomarker, and survival. We selected original articles written in English about endometrial cancer, molecular biomarkers, and that included survival analysis published between January 2000 and December 2016.

**Results:**

Several molecular prognostic biomarkers have been studied in terms of survival and therapeutic response in women with endometrial cancer; hormone receptors, microRNAs, and other molecules have emerged as potentially useful biomarkers, including HER2, p21, HE4, PTEN, p27, ANCCA, and ANXA2.

**Conclusions:**

The use of biomarkers in the assessment of OS, RFS, and CSS requires large trials to expand our understanding of endometrial carcinogenesis. Several molecular markers are significantly associated with a high tumor grade and advanced clinical stage in EC and, therefore, could have additive effects when combined.

## Introduction

Endometrial cancer (EC) is the fourth most common malignancy in women worldwide and the most common gynecological cancer in developed countries. It accounts for up to 30% of malignant gynecologic tumors and has a 5-year survival rate of 84% for white women, but only 62% for black women [1–3]. According to the Global Cancer Observatory (formerly GLOBOCAN) [4] and recent reports, the incidence of EC is 10-15 cases per 100,000 women, and 90% of cases are sporadic [5]. In 2018, 319,605 new cases were estimated worldwide [6], It is the fourth most commonly diagnosed cancer and the seventh most common cause of cancer death among American women. New uterine cancer cases, corresponding to 27 cases per 100,000 women, were reported in the United States, and 10,733 uterine cancer deaths (five deaths per 100,000 women) were reported in 2016 [1]. The increasing trend in incidence has been attributed to a longer expected life span and a higher frequency of overweight and obesity in developed countries [6, 7]. Approximately 25% of women diagnosed with EC have a history of precursor lesions such as endometrial hyperplasia, a known risk factor for invasive disease [8]. In general, most cases are low-risk lesions confined to the uterus (70%), and 80-90% of cases show endometrioid histology, in which an excellent prognosis is expected; nonetheless, 15-20% of patients experience disease recurrence within few years after diagnosis [9]. Advanced disease is associated with high morbidity and mortality, with a survival rate of 16-67% and a mean survival time of 8 to 16 months. Advanced disease also shows high failure rates in response to adjuvant therapies [10].

Early diagnosis of EC is usually achieved as a consequence of the pivotal symptom of abnormal uterine bleeding in 90% of cases [11]. Diagnostic procedures include the measurement of endometrial thickness, endometrial sampling, ultrasonography, hysteroscopy, and dilatation and curettage [12,14], with histology as the cornerstone for diagnosis. Early diagnosis (e.g., tumor stage IA and grades 1 or 2) of EC is associated with a favorable prognosis [13]. The most relevant prognostic factors at diagnosis include the stage, grade, depth of invasive disease, lymphovascular space invasion, and histologic subtype. Patients with endometrial endometrioid tumors have a 5-year survival rate of 83% compared with 62% for those with clear-cell tumors and 53% for endometrial serous carcinomas, also known as uterine papillary serous carcinomas. The 5-year survival rate is 64% in the case of lymphovascular invasion and 88% in its absence [12,15].

Based on the high mortality and morbidity associated with advanced EC, it is crucial to discover new prognostic biomarkers. Many potential molecular biomarkers studied in the last three decades have been associated with prognosis. In a study conducted by Townsend and colleagues, 589 patients were studied to identify differential gene expression. They proposed that the jagged canonical Notch ligand 2 (JAG2), Aurora kinase A (AURKA), phosphoglycerate kinase 1 (PGK1), and hypoxanthine phosphoribosyltransferase 1 (HRPT1) could be used independently as diagnostic, prognostic, or treatment biomarkers in endometrial cancer [16]. Very recently, the results derived from the Cancer Genome Atlas have defined four groups of prognosis based on molecular classifiers [17]: POLE, ultra-mutated, MSI hyper mutated, copy number (CN) low, and CN high [18]. These classifiers correlate with progression-free survival, but are still far from being implemented in clinical practice, but they will also be commented on in this review.

Other studies have demonstrated the benefits of assessing overall survival (OS), cancer-specific survival (CSS), and relapse-free survival (RFS). Clinical and histopathological parameters, such as histologic grade and the International Federation of Gynecology and Obstetrics (FIGO) staging, are prognostic factors [19]. In this review, we will describe the most relevant evidence regarding selected potential molecular biomarkers that may predict OS, RFS, and CSS in EC. We will also describe the molecular pathways related to these biomarkers and their contribution to endometrial carcinogenesis and progression.

## Materials and methods

An exhaustive search was performed in the most important medical database, PUBMED, which is part of the National Library of Medicine of the National Institutes of Health; we used the search terms endometrial cancer, molecular biomarker, and survival. The selection criteria were as follows: original articles, written in English about endometrial cancer, molecular biomarkers, and that included survival analysis published between January 2000 and December 2016.

## Results

We identified 2040 articles, of which 98 articles were selected for this review and separated by molecular bases and clinical implications. We also added information about the biomarkers whether they were blood or tissue-based.

### Blood biomarkers in EC

#### CA-125

High CA-125 serum levels have been associated with myometrial invasion, extrauterine propagation, positive peritoneal cytology, lymph nodes metastasis, recurrence, advanced stage, and lower survival in EC [20]. He et al. evaluated 254 patients with endometrial alterations (proliferativephase,secretoryphase,functionalendometrial polyps, simple hyperplasia, complex hyperplasia, and EC) in a 5-year follow-up study. In 126 women with EC of histologic grades 1 and 2, CA-125 serum levels were significantly higher in comparison with healthy women or with hyperplasia (18.98±2.76 U/ml vs 43.12±13.58 U/ml, respectively), while women with complex hyperplasia showed similar CA-125 levels. Additionally, women with histologic grade 3 EC showed higher levels of CA-125 than healthy women or with simple hyperplasia, but significantly lower levels when compared with those with grades 1 and 2 EC (26.77±3.64 in grade 3) [21]. Zhou et al. [22] reported a mean CA-125 level of 22.99 U/ml in patients with atypical endometrial hyperplasia, and 62.04 U/ml in women with EC. They established a cutoff value of 14.30 U/ml and obtained a sensitivity of 70% and a specificity of 64% as a predictor of EC. This group has also suggested to perform hysterectomy when the CA-125 level is ≥14.3 U/ml, CA-199 is ≥14.06 U/ml and when patients are older than 51.5 years. For younger patients, Zhou et al. proposed to consider uterine depth invasion, endometrial thickness, age at menarche and menopause, parity, and body mass index before performing a hysterectomy [22]. After evaluating 106 Japanese patients with EC, Nakamura et al. reported that high CA-125 plasma levels were associated with an advanced FIGO stage [23]. Additionally, in a retrospective study that included 282 Chinese patients, Li et al. identified a significant association between CA-125 serum levels and the FIGO staging [24].

The role of CA-125 serum levels as an independent predictor of survival is unclear [25]. A previous study found that 21.4% of patients with endometrioid EC have CA-125 serum levels >35 U/ml. In addition, preoperative CA-125 levels between 16.2 and 40.8 U/ml have been associated with a poor prognosis in endometrioid EC [25]. Kim et al. suggested that values above 70 U/ml might be useful to predict OS [26]. Furthermore, Chao et al. proposed that CA-125 levels above 35 U/ml in patients older than 49 years and above 105 U/ml in patients younger than 49 years could predict CSS in EC [27]. Chen et al. evaluated if CA-125 levels predicted a poor prognosis in EC. Their results revealed that 5-year OS and RFS are significantly higher in patients with CA-125 serum levels ≤40 U/ml without lymph node metastasis than in those with serum levels >40 U/ml and lymph nodes metastasis [28]. Additionally, RFS in patients with CA-125 ≤25 U/ml is usually better than in those with levels >25 U/ml. According to these studies, at 25 U/ml, CA-125 could be a useful cutoff value for predicting lymph node invasion and determining whether to perform a lymphadenectomy in patients in early clinical stages of EC [20]. Nonetheless, other authors have suggested that lymphadenectomy should be performed when CA-125 >40 U/ml [12, 28]. However, a cutoff value of 40 U/ml CA-125 could be useful for predicting EC recurrence (sensibility 58.3%, specificity 77.8%), and the association between clinical stage and serum levels of CA-125 can help identify patients who require adjuvant therapy or clinical follow-up [28].

CA-125 serum levels >30 U/ml have been proposed to be useful in determining the adnexal involvement in patients with EC (sensitivity 84.6%, specificity 84.3%) [29]. Additionally, the values above this threshold (30 U/ml) have been associated with extrauterine micro-metastases (lymph nodes, distant metastasis, and positive peritoneal cytology) in early stages (clinical stage I: sensitivity 74.3%, specificity 81.9%). These results suggest that CA-125 could be useful for identifying patients who could benefit from complete tumor debulking [29]. To predict pelvic lymph node metastasis, Yoon et al. have suggested a cutoff value of 31 U/ml, especially to identify patients with para-aortic lymph node involvement (sensitivity 83.3%, specificity 76.8%) [30]. Jiang et al. proposed that CA-125 serum levels above 25 U/ml have a sensitivity and specificity of 78% to predict lymph node metastasis [20].

**Figure 1:**
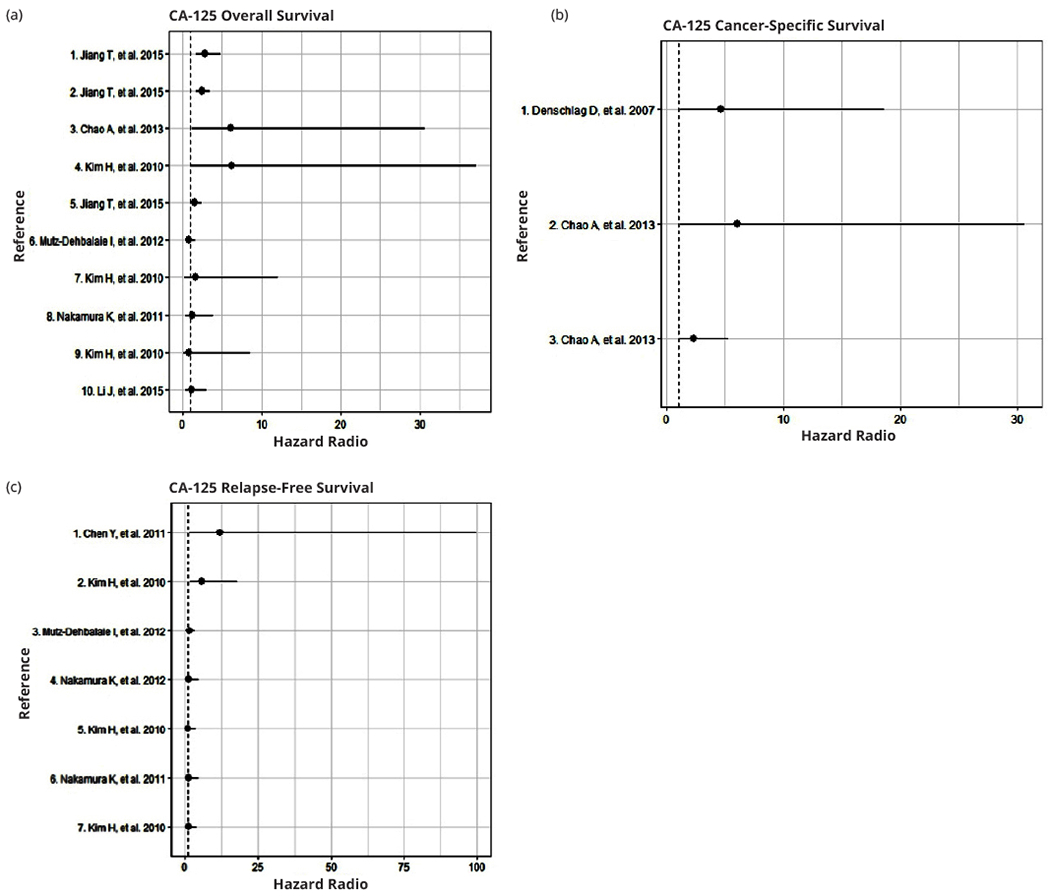
Forest plots of Hazard Ratios for the association of high CA-125 with: (a) Overall survival; (b) Cancer-specific survival; and (c) Relapse-free survival

CA-125 serum levels have been widely studied in several settings to assess prognosis and as a biomarker for treatment response, FIGO stage, and for surgical decisions (i.e., the performance of a lymphadenectomy, which will affect survival). The evidence suggests that CA-125 might be a valuable molecular biomarker with clinical implications in EC. However, combining CA-125 measurements with other biomarkers (e.g., human epididymal secretory protein E4 [HE4]) could improve the prognostic value in EC. From our perspective, CA-125 levels should be determined during the initial approach to EC patients with advanced stage disease, during pre-surgical evaluation and postoperative follow-up, especially because of the relationship between high CA-125 serum levels and post-surgical invasion and metastasis [26, 30]. Although 35 IU/ml has been proposed as a cutoff level in EC patients, other studies have suggested that 20-25 IU/ml at EC diagnosis is significantly associated with clinical stage and prognosis [20, 26,27]. A summary of clinical studies evaluating the impact of CA-125 on OS, CSS and RFS are shown in Figure 1 (Supplementary Table 1).

### Tissue biomarkers in EC

#### Estrogen receptor

**Figure 2:**
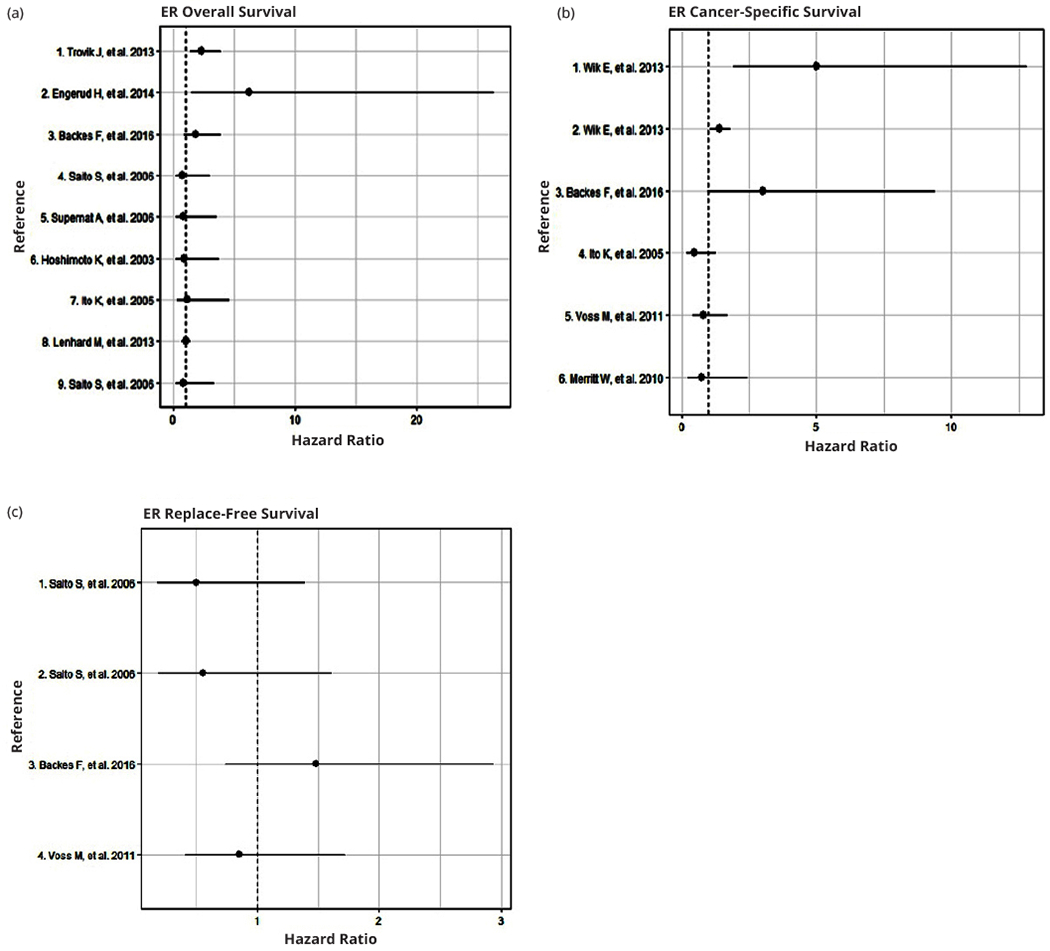
Forest plots of Hazard Ratios for the association of high estrogen receptors levels with: (a) Overall survival; (b) Cancer-specific survival; and (c) Relapse-free survival.

Estrogen exposure is a well-known major risk factor for EC (~80% of EC cases are estrogen-dependent) [31]. ER*α* expression is involved in epithelial-mesenchymal transition (EMT), which is related to myometrial invasion and metastasis due to the contribution of cell migration. The ER*α*-negative condition [ER(−)] is present in 21% of endometrioid cases and has been associated with advanced stage and reduced survival [32]. Low ERα expression has also been associated with EMT and PIK3CA alterations, which may have implications for the choice of adjuvant therapy and targeted agents; therefore, there is a possibility that ERα expression has prognostic value in EC [33]. Some retrospective studies indicate that the ER status in the primary tumor is an independent prognostic marker in EC [34]. In a recent study, a high level of heat-shock factor 1 (HSF1) in ERα-positive tumors was associated with a non-endometrioid type, high grade, and aneuploidy; and it negatively impacted CSS [35]. Backes et al. reported that ER(−) status was found in 18.6% of EC tumors and was associated with an advanced stage and lymph node metastasis [32]. However, ER(−) status did not predict OS, CSS, or RFS [32]. Wik et al. also found that ER(−) status was associated with EMT and reduced CSS [33]. However, other studies have shown that ER status is not significantly associated with survival [36, 37]. This evidence suggests that although a large number of EC tumors are estrogen-dependent, those lacking ER expression are associated with worse survival, which could drive therapeutic decisions, including surgical procedures. A summary of clinical studies evaluating the impact of ER on OS, CSS, and RFS is shown in Figure 2 (Supplementary Table 2).

#### Progesterone receptor

Progesterone is considered to antagonize estrogen-mediated cell proliferation and to induce cell differentiation. whereas loss of progesterone receptor (PR) expression is related to endometrioid carcinoma (especially PR-A). Downregulation [PR(−)] of PR-A, PR-B, or both isoforms has been reported in EC [38]. A recent study conducted by Gates et al. suggested that PR(−) EC tumors are associated with advanced stage, high grade, and deep myometrial invasion [39]. In contrast, Supernat et al. showed that PR(+) status was associated with shorter OS [40]. However, other studies have found that PR expression is not directly related to survival prediction [36, 41]. Additional studies are required to determine the clinical relevance of PR as a predictor of EC patient prognosis, especially because of the controversial results derived from studies with limited sample size. A summary of clinical studies evaluating the impact of PR on OS, CSS, and RFS is shown in Supplementary Figure 1 (Supplementary Table 3).

#### Gene of human epidermal growth factor 2 (HER2)

In patients with EC showing high levels of ER and PR, favorable survival can be predicted; in contrast, when the gene of human epidermal growth factor receptor 2 (HER2) levels rise, the worst survival is observed due to the development of aggressive tumor characteristics [42, 43]. Most epithelial cases are marked by functional activation of growth factors and amplification of the epidermal growth factor receptor HER2. Mutations, such as TP53 mutation, have been demonstrated to be crucial for tumor aggression potential and therapeutic resistance because they promote alterations in transcription [44]. Therefore, the differentiation of cancer subtypes and overexpression of HER2 as a prognostic marker could help predict treatment response and survival [45].

Growdon et al. identified HER2 expression in 59% of endometrial tumors using immunohistochemistry.HER2 gene amplification was evidenced in 18% of tumors; additionally, high expression levels of the variant p95HER2 significantly correlated with high-grade endometrial carcinoma [46]. A correlational study in which hormone receptors and HER2 were evaluated showed that ER−/PR−/HER2+ subtypes exhibited aggressive characteristics, while ER+/PR+/HER2− subtypes showed not so aggressive phenotypes. In addition, ER−/PR−/HER2+ subtypes resulted in a shorter OS, which indicates that high HER2 expression levels are associated with poor survival and aggressive tumor behavior [43]. Furthermore, Gates et al. found an association between HER2+, low body mass index (BMI) and advanced disease stage, which resulted in shorter survival [39]. Nonetheless, Voss et al. concluded that HER2 is not a prognostic molecular biomarker for survival [36]. While ER expression in EC has been widely studied, in contrast to the lack of evidence regarding the use of PR as a biomarker, correlational studies including expression profiles of ER, PR, and HER2 are more useful than the isolated determination of each molecular biomarker for the categorization of aggressive tumors and survival prediction. A summary of clinical studies evaluating the impact of HER2 on OS, RFS, and CSS is shown in Table 1.

#### Tumor protein 53 (TP53)

The study conducted by Singh et al. showed that TP53 was more commonly expressed in African-American than in Caucasian women with EC. In that study, the 5-year survival rate dropped from 85% in the TP53-normal expression group to 52% in the TP53-overexpressed group [47]. In contrast, Trovik et al. reported an abnormal pathologic TP53 expression in 24% of EC samples, which was associated with an older age at diagnosis, advanced stage, lymph node metastasis, non-endometrioid subtype, and advanced histologic grade. Their study also showed that aberrant TP53 expression associated with ER/PR(−) expression predicted poor RFS [34]. However, Voss et al. reported that TP53 alone was not a significant predictor for prognosis in EC [36]. A summary of clinical studies evaluating the impact of TP53 on OS, CSS, and RFS is shown in Supplementary Figure 2 (Supplementary Table 4).

#### Other potential biomarkers in EC

**Figure 3:**
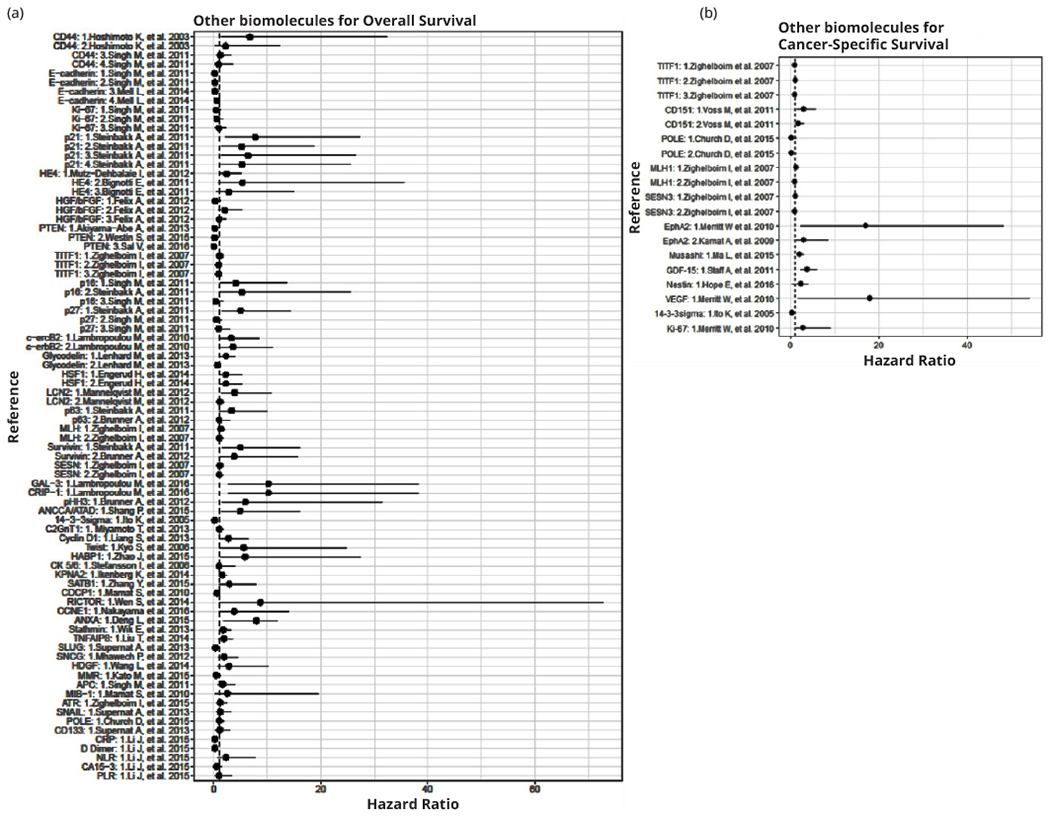
Forest plots of Hazard Ratios for the association of different biomolecules with: (a) Overall survival; (b) Cancer-specific survival.

Other biomarkers have been proposed to assess OS (Figure 3, Panel A), CSS (Figure 3, Panel B), and RFS; however, additional studies are needed to confirm their clinical value, as shown in Supplementary Table 5.

##### CD44 molecule (Indian blood group):

The expression of CD44 has been studied in various carcinomas and was correlated with metastatic potential. In a study performed by Hoshimoto et alCD44 expression was associated with cancer development, invasion, and metastasis [48].

##### E-cadherin:

Reduced E-cadherin expression has been associated with many types of cancer. E-cadherin alterations and its associated cytoplasmic proteins may play a role in determining differentiation in endometrial carcinoma. The role of E-cadherin in the genesis of metastasis and, consequently, a worse prognosis is explained by the relationship between its decreased expression and poor differentiation of non-endometrioid tumors [49]. The inactivation of E-cadherin has been shown in 80-90% of cases of high-grade EC characterized by progressive behavior and deep myometrial invasion [50].

##### Cyclin-dependent kinase inhibitor 1 (p21):

Low p21 expression contributes to increased proliferation and hence, a poor prognosis [51]. Steinbakk et al. studied several endometrial curettage samples of FIGO stage I endometrial endometrioid adenocarcinoma and evaluated the expression of p21 and survivin. They found that low p21 and high survivin expression levels are related to poor prognosis [51].

##### Human epididymal secretory protein E4 (HE4):

HE4 overexpression in EC cells has been observed during both *in vivo* and *in vitro* cell proliferation [52]. Increased HE4 serum levels in EC patients have been strongly suggested to be monitored for evaluation of EC recurrence. Changes in plasma HE4 in association with CA-125 levels during follow-up have been shown to predict EC recurrence, predominantly in patients with endometrioid histology [53]. In patients with EC, HE4 is significantly upregulated compared with normal endometrium [54]. Moore et al. reported the superiority of HE4 over CA-125 for early detection of EC, especially in early stages [55]. In healthy premenopausal women, HE4 assessments increased the sensitivity of CA-125 without compromising its specificity. In some cases, HE4 levels are increased even when CA-125 is not detected, which may contribute to evaluate the therapeutic response or detect early recurrence [56]. Kalogera et al. reported that HE4 has a higher specificity than CA-125 to predict advanced stages of EC. The authors also described that higher levels of CA-125 and HE4 were significantly associated with more aggressive tumors, while the combination of both biomarkers was associated with a shorter OS [57]. Higher HE4 levels were also associated with a higher FIGO stage and histologic grade, as well as with the depth of invasion, preoperative level of CA-125, residual tumor, and platinum resistance [58, 59]. Other authors have suggested that HE4 could be useful for identifying patients who are good candidates for pelvic and para-aortic lymphadenectomy before surgical procedures [60]. Brennan et al. demonstrated that elevated HE4 expression is an independent predictor of RFS in endometrioid subtypes [61]. HE4 is less frequently elevated in benign disease compared with CA-125 (8% vs 29%). The combination of CA-125 and HE4 has been tested as a predictor of malignancy in EC when age is added to the model.

##### Hepatocyte growth factor (HGF) and basic fibroblast growth factor (bFGF):

Felix et al. reported that basic fibroblast growth factor (bFGF) is associated with poor OS and poor RFS. Cases with HGF-positive, stromal bFGF-negative tumors present a lower risk of death compared with cases with HGF-positive, bFGF-negative tumors. Cases with both HGF- and bFGF-positive tumors have a higher risk of recurrence than those with negative expression of both biomarkers [62].

##### Phosphatase and tensin homolog *(PTEN):*

Loss of PTEN has been identified as an independent prognostic marker for favorable survival in endometrial carcinoma. The percentage of cases with loss of PTEN is remarkable in EC compared with patients with atypical endometrial hyperplasia, in which there is also a high association with miR-200c, and PTEN expression is usually negative [63]. Changes related to PTEN occur early during carcinogenesis, developing from latent precancerous lesions [64]. Additionally, there is evidence of an interaction between PTEN and other key cell regulatory proteins contributing to tumor invasion, such as Paired box protein (PAX2) [64, 65]. PTEN has been reported as linked with other genes, including SCUBE2 (Signal Peptide, CUB Domain and EGF Like Domain Containing 2) transcription, as well as with ER and PR [66]. SCUBE2, in association with ER and PR, plays an important role in the clinical prognosis of EC. In endometrioid carcinoma, PTEN mutations are associated with other mutations found in KRAS proto-oncogene, Catenin Beta 1 (CTNNB1), and PIK3CA genes and microsatellite instability, which results in short DNA sequence alterations and its ultimate replication [44, 67, 68]. PTEN sub-expression rates, as well as key genes in the PI3K and β-catenin pathways, are positively related to myometrial invasion in endometrioid carcinoma. PTEN is also associated with increased myometrial invasion, but nonetheless, PTEN and β-catenin overexpression show no significant association with 5-year OS or RFS; thus, the association of PTEN and β-catenin with long-term survival remains to be determined [69]. Obesity is a risk factor related to EC due to PI3K pathway and insulin signaling mutations, which occur in most endometrioid adenocarcinomas. In 187 patients diagnosed with EC, Westin et al. found a relationship between BMI and loss of PTEN, which indicated an increased RFS, including obese patients with a BMI ≥30. Loss of PTEN results in changes in the activation of PI3K pathway proteins, and thus indicates a better prognosis for patients with obesity and loss of PTEN. Akiyama et al. analyzed PTEN expression in 221 endometrial carcinomas; they revealed that the loss of PTEN in histologic grade 1 tumors, according to the FIGO classification, and the absence of lymphovascular invasion were associated with increased survival [70].

##### Heat shock factor (HSF1):

In a study conducted by Engerud et al., high levels of HSF1 were associated with aggressive disease and poor survival (HR=2.3, CI=1.0-5.3, p-value=0.04, n=823). These findings could lead to evaluate HSF1 as a molecular biomarker of OS in EC; however, additional studies are needed to determine its clinical value [35].

##### Microsatellite instability *(MSI) and mismatch repair protein MLH1.*

A study conducted by Zighelboim et al. evaluating MSI and MLH1 methylation status showed that MSI+ tumors without MSH1 methylation were associated with younger patients; nonetheless, MSI was not associated with OS or RFS, and a combined MSI/MSH1 methylation status did not predict OS. These findings indicate that MSI is not associated with survival in patients with endometrioid EC [51, 71].

##### Cyclin-dependent kinase inhibitor 2A, CDKN2A or p16:

Two studies conducted by Singh et al. and Steinbakk et al. showed the clinical relevance (HR=4.18, CI=1.28-13.6, p-value=0.018, n=42; HR=4.7, CI=1.0-21.1, p-value=0.03, n=224; respectively) of negative p16 expression in the assessment of stage IV or recurrent EC with reduced survival and an increased risk of death [47, 51 ].

##### Glycodelin:

Lenhard et al. examined a clinical cohort to determine the role of glycodelin and glycodelin A (GdA) in the evaluation of survival in EC patients, most of whom were in early stages of the disease and had endometrioid histology. The authors found that high levels of glycodelin are associated with a favorable prognosis (HR=0.74, CI=0.45-1.20, p-value=0.232, n=292). In contrast, high GdA is associated with poor clinical outcomes (HR=2.31, CI=1.36-3.94, p-value=0.002, n=292) [72].

##### Lipocalin 2 (LCN2):

Increased lipocalin 2 expression is associated with aggressive features, distant metastases, and poor prognosis in EC. In contrast, the absence of LCN2 showed an association with improved survival. Moreover, strong LCN2 expression (poor prognosis) was associated with ER− and PR-negative tumors.

##### Tumor Protein P63:

Loss of p63 expression is associated with tumor progression and decreased survival. Previous reports have described p63 expression almost exclusively in the endometrioid subtype and loss of p63 in myometrial infiltration [73]. In studies conducted by Steinbakk et al. and Steffansson et al., the absence of p63 expression was associated with non-endometrioid carcinomas, high histologic grade, and reduced patient survival (HR=3.3, CI=1.1-9.9, p-value=0.02, n=224; HR=1.0, CI=0.5-2.9, p-value=0.9, n=76; respectively). Nonetheless, additional studies are required to elucidate the role of p63 in the evaluation of survival and development of EC [51, 74].

##### Survivin:

In confirmed FIGO stage I-IIA endometrial endometrioid carcinomas, using survivin, p21, and p53 as combined biomarkers has a stronger prognostic value than classical parameters, either alone or combined [51 ].

##### Protein GAL3 and Cysteine-rich intestinal protein 1.

Higher GAL3 levels are correlated with tumor progression EC patients. In contrast, cysteine-rich intestinal protein 1 (CRIP1) can modify cytokine patterns and the immune response. In a study conducted by Lambropoulou et al., a correlation was found between GAL3 and CRIP1 by determining the immunohistochemical expression level, and high GAL3 expression was associated with the non-endometrioid histologic type. Moreover, CRIP1 expression is associated with stage III disease. According to the survival analysis, the mean survival time decreases with the increasing expression of GAL3 or CRIP1. Low to moderate GAL3 expression is related to a zero incidence of death, while high expression is related to an 88.9% incidence of death [75].

##### Phosphohistone H3:

Brunner and colleagues analyzed the expression of phosphohistone 3 (pHH3) and reported that patients with high grade endometrioid tumors and carcinomas expressed significantly higher levels of pHH3 and survivin than those with low-grade tumors. pHH3 and survivin were both associated with carcinomas and high grade tumors (p<0.001). The authors concluded that increased pHH3 and survivin expression levels were associated with adverse prognostic factors [76].

##### *AAA* nuclear coregulator cancer-associated *protein (ANCCA):*

In samples from 207 patients with EC and 85 controls, Shang et al. showed that ANCCA was overexpressed in EC patients compared with those with normal endometrium (p<0.001). Furthermore, high expression was associated with significantly poorer OS and CSS than low expression. Through multivariate analysis, high ANCCA expression was an independent factor for OS and CSS [77].

##### *Glucosaminyl (N-acetyl) transferase* 1 (*C2GnT1):*

A short study (n=84) conducted by Miyamoto et al. evaluated C2GnT1 expression using immunohistochemistry in paraffin-embedded endometrial tissues. High C2GnT1 expression correlated with high grade and advanced stage tumors [78].

##### Cyclin D1:

Using immunohistochemistry analysis of 201 EC samples, Liang et al. observed that patients with high Cyclin D1 expression had poorer prognosis than patients without such expression. Additionally, high Cyclin D1 expression was observed in metastasis. Nonetheless, Liang et al. observed that Cyclin D1 poorly differentiate neoplastic lesions from non-neoplastic lesions, and thus it is not applicable for differentiation between benign and malignant lesions [79].

##### Hyaluronic acid binding protein 1 (HABP1):

HABP1, which was studied by Zhao et al., is involved in tumorigenesis, progression, invasion and metastasis. HABP1 was overexpressed in EC and benign endometrial lesions compared with normal endometrium (significantly higher in EC) and associated with recurrence and poor OS and RFS, which indicates that HABP1 overexpression might serve as a new biomarker [80].

##### Tumor necrosis factor-a-induced protein 8 (TNFAIP8):

Liu et al. reported that overexpression of TNFAIP8 is associated with an advanced FIGO stage (p<0.001), higher histologic grade (p=0.017), myometrial invasion (p=0.030), lymphovascular space invasion (p=0.011), lymph node metastasis (p<0.001,) and recurrence (p=0.002) [81].

##### Karyopherin subunit alpha-2 (KPNA2):

Ikenberg et al. showed that KPNA2 expression is upregulated in endometrial carcinoma. Additionally, nuclear KPNA2 immunoreactivity was identified as a predictor of OS. Nonetheless, no association between KPNA2 expression and EC subtype has been detected [82].

##### CUB domain-containing protein 1 (CDCP1):

Mamat et al. examined CDCP1 expression levels in endometrioid carcinoma and observed a positive correlation between low CDCP1 expression and stage, relapse rate, and poor prognosis. Low CDCP1 expression and advanced stage have also been demonstrated to be independent poor prognostic factors for OS and RFS [83].

##### CCNE1 (cyclin E):

CCNE1 amplification has been observed to be significantly correlated with shorter OS and RFS. In a study performed by Nakayama et al., CCNE1 expression was limited to 9 of 108 endometrial carcinomas, and CCNE1 overexpression was associated with reduced OS and RFS compared with an absence of CCNE1 amplification. The authors suggested that CCNE1-targeted therapy could have survival benefits in patients with CCNE1 overexpression [1].

##### Annexin A2 (ANXA2):

Alonso-Alconada et al. studied ANXA2 for its role as a potential molecular biomarker associated with recurrent disease; they highlighted its clinical use as a prognostic predictor in EC. However, larger studies evaluating ANXA2 are needed [9].

##### Stathmin:

In a study conducted by Wik et al. evaluating two series, one assessing the role of stathmin and the other validating the results derived from the first one, high stathmin expression was associated with clinical progress in EC (i.e., poor prognosis, assessed by RFS and CSS, and increased tumor cell proliferation) [84].

##### Hepatoma-derived growth factor (HDGF):

Wang et al. examined the correlation between high HDGF expression and clinical data in EC, including patient survival. High expression was positively associated with FIGO stage but not with other clinical features. High HDGF expression, unlike low expression, was associated with lower OS rates. These findings suggest that high HDGF expression is a potential unfavorable prognostic factor for the progression and prognosis of EC [85].

##### CD151 (CD151 Molecule (Raph Blood Group)):

Voss et al. found that low CD151 expression was associated with significantly worse CSS and RFS, as opposed to high expression. In contrast, after analyzing other molecular biomarkers (ER, PR, p53, and HER2), they concluded that these proteins were not significant factors influencing survival and thus, they contradicted previous findings [36].

##### Receptor tyrosine kinase (EphA2):

Using immunohistochemistry in 139 EC samples, Kamat et al. examined the expression of EphA2, ER, PR, and Ki-67. EphA2 was associated with high stage, high grade, increased myometrial invasion depth, low ER and PR expression, and high Ki-67 expression. Furthermore, as indicated previously, lack of ER and PR expression are both associated with high grade and lymph node metastasis. In conclusion, EphA2 overexpression is associated with poor outcomes and reduced survival and may be an important therapeutic target, especially in patients with ER− and PR− negative EC.

##### Musashi:

By analyzing 35 fresh EC samples and 15 fresh normal endometrial samples, as well as 148 paraffin-embedded EC tissue samples and 20 paraffin-embedded normal endometrial samples using immunohistochemistry, Ma et al. found that high levels of Musashi-1 protein expression were associated with poor OS in patients with EC [86].

##### Growth differentiation factor 15 (GDF-15):

Staff et al. measured GDF-15 plasma levels in patients with EC and found that median GDF-15 plasma concentrations in the EC group were elevated (1077 ng/l) in comparison with those in the premenopausal and postmenopausal controls (590 and 684 ng/l). High plasma levels were associated with FIGO stage III/IV disease, non-endometroid histology, high grade, older age, postmenopausal status, and lymph node metastasis, all of which affect survival. High GDF-15 levels were also an independent predictor of poor RFS and CSS. However, its applicability as a predictor of node metastasis and in monitoring the treatment of EC must be further studied and compared in larger studies [87].

##### Heterogeneous nuclear ribonucleoprotein G (hnRNP G) and protein hTra-β1:

Ouyang et al. evaluated two molecular biomarkers, the tumor suppressor hnRNP and hTra2-p1, and found a prominent expression of hnRNP G in patients without metastases and in early stages of disease. The levels of hTra-β1 were increased in poorly differentiated malignancies and lymph node metastasis. An elevated hnRNP G level was associated with favorable outcomes. In addition, a multivariable analysis revealed that hnRNP G and hTra-β1 were independent factors for RFS in EC [88].

##### CDK4/6-cyclin D:

CDK4 is elevated in 34-77% of endometrioid EC cases and is considered to be an early event in neoplastic transformation in EC. Although CDK6 interacts with CDK4, its role has not been clearly determined. However, Ikeda et al. examined CDK4 and CDK6 in the assessment of RFS in EC. CDK4/6 were useful molecular biomarkers for predicting prognosis (CI:1.34-86.87, p=0.026). This biomarker is higher in low-risk patients than in intermediate or high-risk patients, which shows that patients with high CDK 4/6 expression have shorter RFS than those with lower expression. Additionally, it may be useful for the prediction of tumor chemosensitivity. Nonetheless, additional studies are required [89].

##### Gamma-glutamyl transferase GGT

Seebacher et al. described that elevated GGT serum levels were independently associated with RFS in a multivariate analysis. Quantifying GGT prior to therapy was not associated with advanced stage, high grade, or unfavorable histologic subtype [90]. We have summarized the results of clinical studies evaluating the impact of other potential biomolecules on OS (Supplementary Table 5), CSS (Supplementary Table 6), and RFS (Supplementary Table 7) in EC.

##### Proliferation marker Ki-67:

Ki-67 plays a role in tumor formation by controlling cell proliferation [47] since the removal of Ki-67 protein using antisense nucleotides prevents cell proliferation [91]. High Ki-67 expression has been found in various endometrial carcinoma types and is correlated with the histologic grade, depth of myometrial invasion, and risk of recurrence [38].

### New TCGA classification of EC and its prognostic implications

Based on the Cancer Genome Atlas (TCGA), research teams have developed molecular classifiers that identify four new distinct molecular subgroups: POLE ultra-mutated, MSI hypermutated, copy number (CN) low, and CN high, which correlate with progression-free survival. This new classification was based on a combination of whole genome sequencing, exome sequencing, microsatellite instability (MSI) assays, and copy number analysis. POLE gene (DNA polymerase epsilon, catalytic subunit) encodes the major catalytic and proofreading subunits of a DNA polymerase enzyme complex, responsible for leading strand DNA replication. The correct exonuclease proofreading function and the high-fidelity incorporation of bases by POLE ensure a low mutation rate in the new replicated strands. Mutations in DNA polymerases inactivate or suppress proofreading abilities, which in turn increase replicative error rates and result in the ultra-mutated phenotype, and therefore contribute to a worse prognosis in EC [18]. MSI arises from defects in the post-replicative DNA mismatch repair system. MSI deficiencies have been previously described in inherited cancer syndromes (e.g., Lynch) and in acquired/somatic mutations or epigenetic events, most commonly involving MLH1 gene (mutL homolog 1). High or low copy number EC is based on significantly reoccurring amplification or deletion regions, determined by bioinformatic approaches. Even though these new molecular findings showed clear prognostic implications, the methodologies used for the TCGA study are costly, complex, and unsuitable for wider clinical applications.

## Discussion

The present study selected and reviewed the most relevant evidence related to the prediction of key clinical and histopathologic features of malignancy in EC but also of OS, RFS, and CSS; it was based on several studies that analyzed many different molecular biomarkers, some of which have been studied and mentioned in multiple articles over the last decades. Other novel molecular biomarkers could have major uses in evaluating EC patient survival; however, a significant number of studies with high standards of methodology and a larger study population are needed.

## Conclusion

The use of biomarkers in assessing OS, RFS, and CSS requires large trials to expand our understanding of EC carcinogenesis and treatment resources. Several molecular markers have been significantly associated with a high tumor grade and advanced clinical stage in EC and, therefore, could have possible additive effects in combination. Accordingly, these molecules could also confer a risk for tumor progression and drug resistance.

## Supplementary Material

Supplementary Material

## Figures and Tables

**Table 1:** HER2 association with overall survival, cancer-specific survival, and relapse-free survival in endometrial cancer.

Reference	Hazard ratio	95% confidence interval	p-value	Median follow-up time (months)	n
Overall survival					
Growdon W, et al. 2015	3.43	1.25-2.24	0.01	17	86
Lapinska-Szumczyk S, et al. 2014	2.07	1.07-4.02	0.03	72	400
Gates E, et al. 2006	4	0.77-20.80	0.1	60	165
Supernat A, et al. 2013	1.37	0.37-5.05	0.64	54.5	156
Cancer-specific survival					
Voss M, et al. 2011	1.54	0.55-4.26	0.402	60	156
Relapse-free survival					
Lapinska-Szumczyk S, et al. 2014	3.49	1.87-6.54	0.002	72	400
Voss M, et al. 2011	1.4	0.51-3.88	0.506	60	156

Numbers in bold=Statistically significant p-values (<0.05)
