# Supplementary material for: Prognostic molecular biomarkers in endometrial cancer: A review
Source: J Cancer Res Ther (Manch). Author manuscript; Available in PMC 2021 Jul 27. (PMC8315102; doi:10.14312/2052-4994.2019-3)
Supplement: Supplementary Material [file NIHMS1653826-supplement-Supplementary_Material.pdf]

Supplementary material

Prognostic molecular biomarkers in endometrial cancer: A review

J. Edgardo Hernández, Ailyn González-Montiel, Jesús C. Ceb Allos-Villalva, David Cantú, Salim Barquet, Anny Olivares-Mundo, Luis A. Herrera, and Diddier Prada

http://dx.doi.org/10.14312/2052-4994.2019-3

Supplementary Tables

Supplementary Table 1 Association between serum CA-125 and overall survival, cancer-specific survival and relapse-free survival in endometrial cancer.

Reference	*Hazard ratio*	*95% Confidence interval*	*p-value*	*Median follow-up time (months)*	*n*
Overall survival					
Jiang T, et al. 2015	2.86	1.71-4.78	**0.001**	35	995
Jiang T, et al. 2015	2.41	1.69-3.46	**0.001**	35	995
Chao A, et al. 2013	6.03	1.19-30.63	**0.03**	53.5	757
Kim H, et al. 2010	6.16	1.02-37.14	**0.04**	26.5	413
Jiang T, et al. 2015	1.54	0.98-2.41	0.06	35	995
Mutz-Dehbalaie I, et al. 2012	0.77	0.38-1.56	0.469	36	183
Kim H, et al. 2010	1.57	0.20-12.07	0.67	26.5	413
Nakamura K, et al. 2011	1.18	0.36-3.80	0.779	47	106
Kim H, et al. 2010	0.83	0.07-8.51	0.83	26.5	413
Li J, et al. 2015	1.07	0.38 - 3.00	0.893	15	282
Cancer-specific survival					
Denschlag D, et al. 2007	4.66	1.17-18.64	**0.029**	39	101
Chao A, et al. 2013	6.03	1.19-30.63	0.05	60	757
Chao A, et al. 2013	2.34	1.04-5.29	0.05	60	757

Relapse-free survival					
Chen Y, et al. 2011	12.07	1.62-99.64	**0.01**	96	120
Kim H, et al. 2010	5.75	1.85-17.89	**0.01**	26.5	413
Mutz-Dehbalaie I, et al. 2012	1.626	0.78-3.37	0.192	36	183
Nakamura K, et al. 2012	1.31	0.37-4.61	0.668	62	111
Kim H, et al. 2010	1.05	0.28-3.89	0.67	26.5	413
Nakamura K, et al. 2011	1.25	0.33-4.62	0.736	46	106
Kim H, et al. 2010	1.29	0.40-4.14	0.94	26.5	413

Bold = Statistically significant p-values (<0.05).

Supplementary Table 2 Association between tumor tissue estrogen receptor (ER) and overall survival, cancer-specific survival and relapse-free survival in endometrial cancer.

Reference	*Hazard ratio*	*95% Confidence interval*	*p-value*	*Median follow-up time (months)*	*n*
Overall survival					
Trovik J, et al. 2013	2.28	1.35-3.86	**0.002**	108	1192
Engerud H, et al. 2014	6.2	1.5-26.4	**0.014**	132	823
Backes F, et al. 2016	1.83	0.86-3.87	0.114	60	323
Saito S, et al. 2006	0.74	0.18-2.99	0.681	60	103
Supernat A, et al. 2013	0.76	0.16-3.55	0.73	54.5	156
Hoshimoto K, et al. 2003	0.85	0.19-3.73	0.83	58.8	175
Ito K, et al. 2005	1.13	0.28-4.59	0.86	60	103
Lenhard M, et al. 2013	0.97	0.65-1.44	0.88	120	292
Saito S, et al. 2006	0.79	0.18-3.36	0.918	60	103
Cancer-specific survival					
Wik E, et al. 2013	5.0	1.9-12.8	**0.001**	108	628
Wik E, et al. 2013	1.4	1.05-1.8	**0.02**	108	628
Backes F, et al. 2016	3.0	0.94-9.39	0.063	60	323
Ito K, et al. 2005	0.46	0.16-1.27	0.135	60	103
Voss M, et al. 2011	0.81	0.38-1.72	0.591	60	156
Merritt W, et al. 2010	0.75	0.23-2.44	0.63	78	85
Relapse-free survival					
Saito S, et al. 2006	0.5	0.18-1.39	0.188	60	103
Saito S, et al. 2006	0.55	0.19-1.61	0.279	60	103
Backes F, et al. 2016	1.48	0.74-2.94	0.368	60	323
Voss M, et al. 2011	0.85	0.41-1.72	0.655	60	156

Bold = Statistically significant p-values (<0.05).

Supplementary Table 3 Association between tumor tissue progesterone receptor (PR) and overall survival, cancer-specific survival and relapse-free survival in endometrial cancer.

Reference	*Hazard ratio*	*95% Confidence interval*	*p-value*	*Median follow-up time (months)*	*n*
Overall survival					
Gates E, et al. 2006	13.9	2.96-65.22	**0.001**	60	165
Supernat A, et al. 2013	0.29	0.10-0.84	**0.02**	54.5	156
Saito S, et al. 2006	0.19	0.02-1.76	0.152	60	103
Saito S, et al. 2006	0.44	0.10-1.93	0.279	60	103
Ito K, et al. 2005	0.43	0.09-2.04	0.29	60	103
Cancer-specific survival					
Ito K, et al. 2005	0.14	0.03-0.51	**0.003**	60	103
Merritt W, et al. 2010	0.46	0.14-1.49	0.18	78	85
Voss M, et al. 2011	0.96	0.34-2.66	0.94	60	156
Relapse-free survival					
Saito S, et al. 2006	0.17	0.03-0.80	**0.025**	60	103
Saito S, et al. 2006	0.37	0.12-1.12	0.079	60	103
Voss M, et al. 2011	0.86	0.31-2.38	0.77	60	156

Bold = Statistically significant p-values (<0.05).

Supplementary Table 4 Association between tumor tissue TP53 and overall survival, cancer-specific survival and relapse-free survival in endometrial cancer.

Reference	*Hazard ratio*	*95% Confidence interval*	*p-value*	*Median follow-up time (months)*	*n*
Overall survival					
Hoshimoto K, et al. 2003	13.67	3.24 - 57.67	**0.01**	58.8	175
Steinbakk A, et al. 2011	2.9	1.5 - 19.2	0.05	209	224
Ito K, et al. 2005	0.33	0.08 - 1.26	0.105	60	103
Singh M, et al. 2011	1.99	0.78 - 5.07	0.15	56	42
Ikenberg K, et al. 2014	1.38	0.87 - 2.18	0.174	240	527
Trovik J, et al. 2013	0.81	0.42 - 1.56	0.531	108	1192
Cancer-specific survival					
Ito K, et al. 2005	0.58	0.17 - 2.02	0.399	60	103
Voss M, et al. 2011	1.28	0.71 - 2.32	0.409	60	156
Relapse-free survival					
Singh M, et al. 2011	1.28	0.53 - 3.07	0.584	56	42
Voss M, et al. 2011	1.11	0.62 - 1.99	0.72	60	156

Bold = Statistically significant p-values (<0.05).

Supplementary Table 5 Other biomolecules in serum and tumors evaluated in different studies for their association with overall survival in endometrial cancer.

Biomolecule	*Hazard ratio*	*95% Confidence interval*	*p-value*	*Median follow-up time (months)*	*n*
CD44					
Hoshimoto K, et al. 2003	6.78	1.42 - 32.33	**0.02**	58.8	175
Hoshimoto K, et al. 2003	2.25	0.41 - 12.29	0.35	58.8	175
Singh M, et al. 2011	1.22	0.48 - 3.11	0.681	56	42
Singh M, et al. 2011	0.91	0.23-3.51	0.886	56	42
E-cadherin					
Singh M, et al. 2011	0.18	0.05-0.59	**0.004**	56	42
Singh M, et al. 2011	0.22	0.07-0.70	**0.01**	56	42
Mell L, et al. 2004	0.23	0.05-0.94	**0.04**	58.5	102
Mell L, et al. 2004	0.59	0.34-1.03	0.066	58.5	102
Ki-67					
Singh M, et al. 2011	0.48	0.18-1.30	0.149	56	42
Singh M, et al. 2011	0.59	0.22-1.59	0.299	56	42
Singh M, et al. 2011	0.97	0.41-2.30	0.947	56	42
p21					
Steinbakk A, et al. 2011	7.8	2.2-27.3	**0.0001**	209	224
Steinbakk A, et al. 2011	5.2	1.5-18.8	**0.005**	209	224
Steinbakk A, et al. 2011	6.4	1.5-26.5	**0.01**	209	224
Steinbakk A, et al. 2011	5.3	1.1-25.5	**0.02**	209	224
*HE4**					
Mutz-Dehbalaie I, et al. 2012	2.4	1.17-4.97	**0.017**	36	183
Bignotti E, et al. 2011	5.35	0.80-35.67	0.08	70	153
Bignotti E, et al. 2011	2.84	0.54-15.01	0.22	70	153
HGF/bFGF					
Felix A, et al. 2012	0.29	0.06-1.33	0.11	144	221
Felix A, et al. 2012	2.09	0.83-5.25	0.12	144	221
Felix A, et al. 2012	1	0.44-2.28	1	144	221
PTEN					
Akiyama-Abe A, et al. 2013	0.21	0.05-0.88	**0.03**	59	221
Westin S, et al. 2015	0.15	0.03-0.9	**0.038**	28.3	242
Sal V, et al. 2016	0.02	0.001-0.32	0.37	59	102

TITF1					
Zighelboim I, et al. 2007	1.07	0.81-1.41	0.622	58.6	930
Zighelboim I, et al. 2007	0.92	0.61-1.40	0.722	58.6	930
Zighelboim I, et al. 2007	0.95	0.70-1.30	0.779	58.6	930
p16					
Singh M, et al. 2011	4.18	1.28-13.6	**0.018**	56	42
Steinbakk A, et al. 2011	5.3	1.1-25.5	**0.02**	209	224
Singh M, et al. 2011	0.39	0.09-1.62	0.196	56	42
p27					
Steinbakk A, et al. 2011	5	1.6-14.4	**0.002**	209	224
Singh M, et al. 2011	0.51	0.18-1.41	0.191	56	42
Singh M, et al. 2011	0.92	0.29-2.95	0.893	56	42
c-erbB2					
Lambropoulou M, et al. 2010	3.28	1.28-8.41	**0.013**	176	110
Lambropoulou M, et al. 2010	3.57	1.17-10.89	**0.026**	176	110
Glycodelin A					
Lenhard M, et al. 2013	2.31	1.36-3.94	**0.002**	120	292
Lenhard M, et al. 2013	0.74	0.45-1.20	0.232	120	292
HSF1					
Engerud H, et al. 2014	2.3	1.0-5.3	**0.04**	132	823
Engerud H, et al. 2014	2.3	1.0-5.3	0.06	132	823
LCN2					
Mannelqvist M, et al. 2012	3.9	1.4-10.8	**0.027**	108	316
Mannelqvist M, et al. 2012	1.1	0.6-1.9	**0.027**	108	316
p63					
Steinbakk A, et al. 2011	3.3	1.1-9.9	**0.02**	209	224
Stefansson I, et al. 2006	1	0.5-2.9	0.9	108	76
MLH1					
Zighelboim I, et al. 2007	1.29	0.84-1.96	0.236	58.6	930
Zighelboim I, et al. 2007	1.04	0.83-1.30	0.701	58.6	930
Survivin					
Steinbakk A, et al. 2011	5	1.6-16.1	**0.002**	209	224
Brunner A, et al. 2012	3.8	1.2-15.6	**0.023**	72	106

SESN3					
Zighelboim I, et al. 2007	1.12	0.84-1.50	0.43	58.6	930
Zighelboim I, et al. 2007	0.99	0.59-1.66	0.992	58.6	930
GAL-3					
Lambropoulou M, et al. 2016	10.24	2.74-38.26	**0.0001**	128	46
CRIP-1					
Lambropoulou M, et al. 2016	10.24	2.74-38.26	**0.0005**	128	46
pHH3					
Brunner A, et al. 2012	5.9	1.7-31.4	**0.004**	72	106
ANCCA/ATAD2					
Shang P, et al. 2015	4.95	1.53-15.96	**0.007**	18	207
14-3-3s					
Ito K, et al. 2005	0.18	0.04-0.71	**0.014**	60	78/103
C2GnT1					
Miyamoto T, et al. 2013	1.04	1.00-1.07	**0.017**	108	48
Cyclin D1					
Liang S, et al. 2013	2.76	1.20-6.36	**0.017**	48	201
Twist					
Kyo S, et al. 2006	5.62	1.27-24.8	**0.023**	39.4	70
HABP1					
Zhao J, et al. 2015	5.84	1.24-27.34	**0.025**	17	272
CK 5/6					
Stefansson I, et al. 2006	1	1.0-3.9	**0.03**	108	115
KPNA2					
Ikenberg K, et al. 2014	1.59	1.04-2.42	**0.031**	240	527
SATB1					
Zhang Y, et al. 2015	2.92	1.07-7.99	**0.036**	22	172
CDCP1					
Mamat S, et al. 2010	0.57	0.33-0.98	**0.04**	113	110
RICTOR					
Wen S, et al. 2014	8.73	1.05-72.70	**0.045**	131	296
CCNE1					
Nakayama K, et al. 2016	3.8	1.0-14.0	**0.045**	151	108

ANXA2 - HE4					
Deng L, et al. 2015	8	1.76-11.96	0.05	108	132
Stathmin					
Wik E, et al. 2013	1.8	1.0-3.1	0.05	115	804
TNFAIP8					
Liu T, et al. 2014	1.88	0.97-3.63	0.059	23	225
SLUG					
Supernat A, et al. 2013	0.34	0.11-1.06	0.06	54.5	156
SNCG					
Mhawech P, et al. 2012	1.97	0.87-4.45	0.1	131	279
HDGF					
Wang L, et al. 2014	2.82	0.78-10.16	0.111	72	122
MMR					
Kato M, et al. 2015	0.49	0.19-1.28	0.14	204	191
APC					
Singh M, et al. 2011	1.65	0.70-3.90	0.254	56	42
ATR					
Zighelboim I, et al. 2015	1.16	0.58-2.32	0.68	48	3838
SNAIL					
Supernat A, et al. 2013	1.15	0.43-3.09	0.78	54.5	156
POLE					
Church D, et al. 2015	1.06	0.59-1.92	0.85	89	788
CD133					
Supernat A, et al. 2013	1.09	0.40-2.92	0.87	54.5	156
*CRP**					
Li J, et al. 2015	0.21	0.08-0.54	**0.001**	15	282
*D dimer**					
Li J, et al. 2015	0.25	0.09-0.67	**0.006**	15	282
*NLR**					
Li J, et al. 2015	2.29	0.67-7.78	0.181	15	282
*CA15-3**					
Li J, et al. 2015	0.53	0.18-1.55	0.25	15	282
*PLR**					
Li J, et al. 2015	0.99	0.29-3.35	0.991	15	282

Bold=Statistically significant p-values (<0.05).

Supplementary Table 6 Biomolecules evaluated in serum and tumors for their association with cancer-specific survival in endometrial cancer.

	Biomolecule	*Hazard ratio*	*95% Confidence interval*	*p-value*	*Median follow-up time (months)*	*n*
TITF1						
	Zighelboim I, et al. 2007	0.89	0.60-1.32	0.574	58.6	930
	Zighelboim I, et al. 2007	1.07	0.812-1.416	0.622	58.6	930
	Zighelboim I, et al. 2007	0.95	0.70-1.30	0.779	58.6	930
CD151						
	Voss M, et al. 2011	2.94	1.50 – 5.74	0.001	60	156
	Voss M, et al. 2011	1.81	1.06-3.10	0.02	60	156
POLE						
	Church D, et al. 2015	0.2	0.03-1.46	0.11	89	788
	Church D, et al. 2015	0.19	0.03-1.44	0.11	89	788
MLH1						
	Zighelboim I, et al. 2007	1.31	0.87-1.96	0.182	58.6	930
	Zighelboim I, et al. 2007	1.04	0.83-1.30	0.7	58.6	930
SESN3						
	Zighelboim I, et al. 2007	1.12	0.84-1.50	0.43	58.6	930
	Zighelboim I, et al. 2007	0.91	0.55-1.49	0.716	58.6	930
EphA2						
	Merritt W, et al. 2010	17.03	2.23-48.28	0.006	78	85
	Kamat A, et al. 2009	3	1.05-8.56	0.04	48	139
Musashi-1						
	Ma L, et al. 2015	2.07	1.37-3.12	0.001	71	168
GDF-15						
	Staff A, et al. 2011	3.72	2.26-6.10	0.001	72	466

Nestin						
	Hope E, et al. 2016	2.32	0.32-4.08	0.003	44	323
VEGF						
	Merritt W, et al. 2010	18.01	1.68-54.22	0.004	78	85
14-3-3s						
	Ito K, et al. 2005	0.32	0.11-0.89	0.029	60	103
Ki-67						
	Merritt W, et al. 2010	2.89	0.92-9.12	0.057	78	85

Bold=Statistically significant p-values (<0.05).

Supplementary Table 7 Biomolecules evaluated in serum and tumors for their association with relapse-free survival in endometrial cancer.

Biomolecule	*Hazard ratio*	*95% Confidence interval*	*p-value*	*Median follow-up time (months)*	*n*
Ki-67					
Liu T, et al. 2014	1.7	0.93-3.09	0.08	23	225
Singh M, et al. 2011	0.82	0.38-1.76	0.615	56	42
Singh M, et al. 2011	0.77	0.28-2.14	0.618	56	42
Singh M, et al. 2011	0.81	0.33-2.01	0.649	56	42
Singh M, et al. 2011	0.87	0.39-1.91	0.723	56	42
*HE4**					
Brennan D, et al. 2014	2.4	1.19-4.83	**0.014**	37	373
Mutz-Dehbalaie I, et al. 2012	1.58	0.82-3.08	0.171	36	183
Bignotti E, et al. 2011	3.18	0.53-19.17	0.21	70	153
Bignotti E, et al. 2011	2.76	0.47-16.14	0.26	70	153
E-cadherin					
Mell L, et al. 2004	0.28	0.10-0.77	**0.014**	58.5	102
Singh M, et al. 2011	0.3	0.10-0.86	**0.025**	56	42
Singh M, et al. 2011	0.44	0.16-1.21	0.112	56	42
ANXA2					
Alonso L, et al. 2015	4.69	2.00-11.03	**0.0004**	60	131
Alonso L, et al. 2015	2.99	1.35-6.64	**0.004**	60	131
Alonso L, et al. 2015	1.71	0.64-4.57	0.28	60	131
HGF/bFGF					
Felix A, et al. 2012	9.88	2.63-37.16	**0.001**	144	221
Felix A, et al. 2012	0.07	0.00-0.81	**0.03**	144	221
Felix A, et al. 2012	1.56	0.44-5.53	0.49	144	221
PTEN					
Akiyama-Abe A, et al. 2013	0.2	0.05-0.86	**0.03**	59	221
Sal V, et al. 2016	0.16	0.01-1.09	0.28	59	102
Westin S, et al. 2015	2.4	0.48-8.61	0.33	28.3	242

CD151					
Voss M, et al. 2011	2.54	1.37-4.71	**0.008**	60	156
Voss M, et al. 2011	11.77	1.06-2.96	**0.02**	60	156
CD44					
Singh M, et al. 2011	2.23	0.85-5.85	0.105	56	42
Singh M, et al. 2011	1.26	0.33-4.81	0.739	56	42
p16					
Singh M, et al. 2011	0.34	0.08-1.36	0.125	56	42
Singh M, et al. 2011	2.02	0.67-6.05	0.209	56	42
p27					
Singh M, et al. 2011	0.35	0.12-1.04	0.058	56	42
Singh M, et al. 2011	0.54	0.17-1.79	0.316	56	42
Nestin					
Hope E, et al. 2016	2.78	1.79-4.32	**0.001**	44	323
hTra2-beta1					
Ouyang Y, et al. 2011	5.76	1.73-19.14	**0.004**	97	139
MMR					
Kato M, et al. 2015	0.24	0.08-0.70	**0.01**	204	191
ANCCA/ATAD2					
Shang P, et al. 2015	4.23	1.29-13.85	**0.017**	18	207
HABP1					
Zhao J, et al. 2015	4.11	1.22-13.85	**0.022**	17	272
CDK4/6					
Ikeda Y, et al. 2015	10.79	1.34-86.87	**0.026**	64	109
hnRNP G					
Ouyang Y, et al. 2011	0.46	0.23-0.91	**0.026**	97	139
CDCP1					
Mamat S, et al. 2010	0.54	0.32-0.94	**0.028**	113	110
SATB1					
Zhang Y, et al. 2015	2.82	1.11-7.18	**0.029**	22	172
TNFAIP8					
Liu T, et al. 2014	2.05	1.07-3.91	**0.029**	23	225
FOLR1					
Allard J, et al. 2007	2.14	1.07-4.28	**0.031**	60	292

FAS					
Sebastiani V, et al. 2003	5.41	0.05-27.85	**0.04**	60	95
POLE					
Church D, et al. 2015	0.43	0.13-1.37	0.15	89	788
APC					
Singh M, et al. 2011	1.92	0.69-5.28	0.21	56	42
CCNE1					
Nakayama K, et al. 2016	2.2	0.6-7.4	0.217	151	108
ATR					
Zighelboim I, et al. 2015	0.61	0.25-1.50	0.28	48	3838
GLUT1					
Sebastiani V, et al. 2003	1.01	0.80-1.27	0.92	60	95
*GGT**					
Seebacher V, et al. 2012	2.1	1.2-3.4	**0.005**	60	874

Bold = Statistically significant p-values (<0.05); *Serum biomarkers.

Supplementary Figures

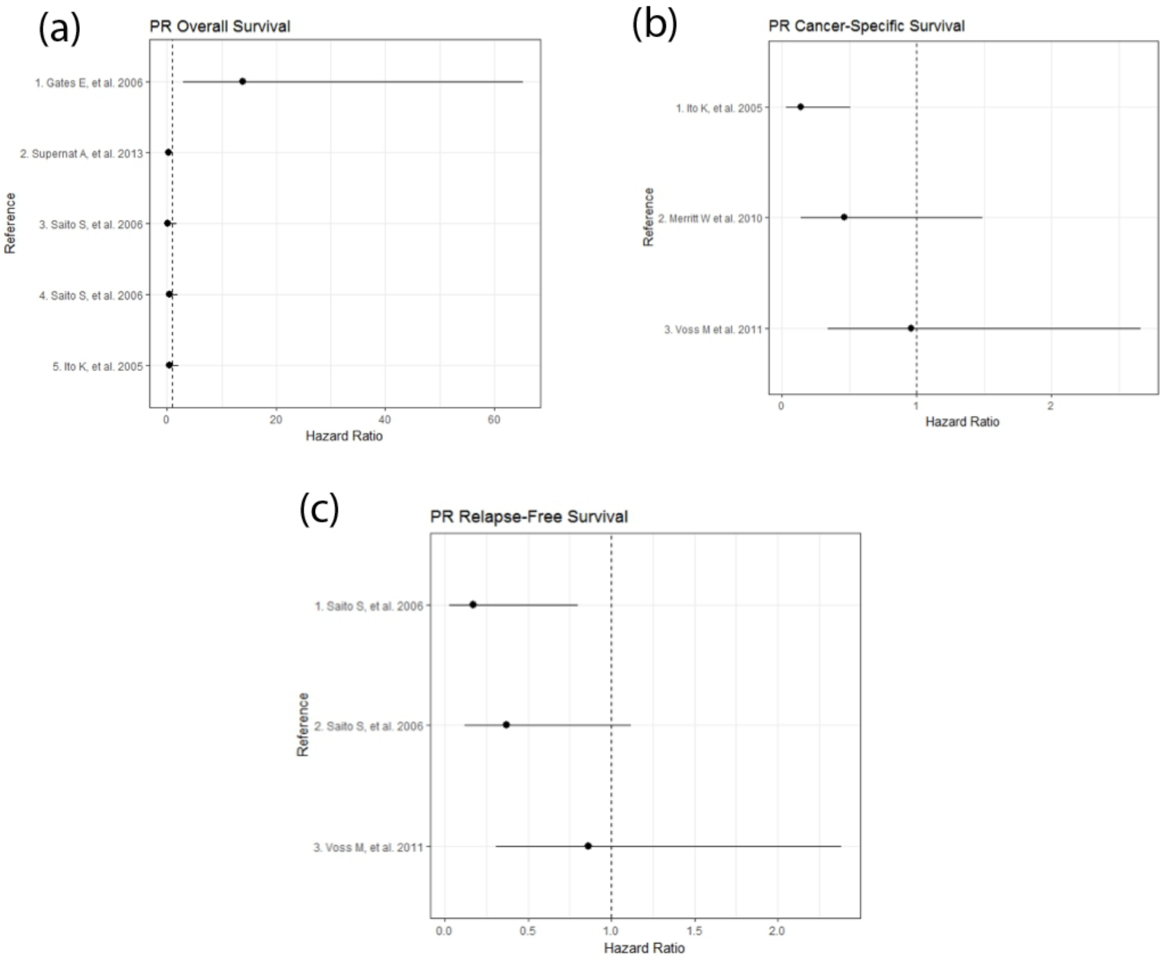

Supplementary Figure 1 Forest plots for the Hazard Ratio of high progesterone receptor for (a) Overall survival; (b) Cancer-specific survival and (c) Relapse-free survival.

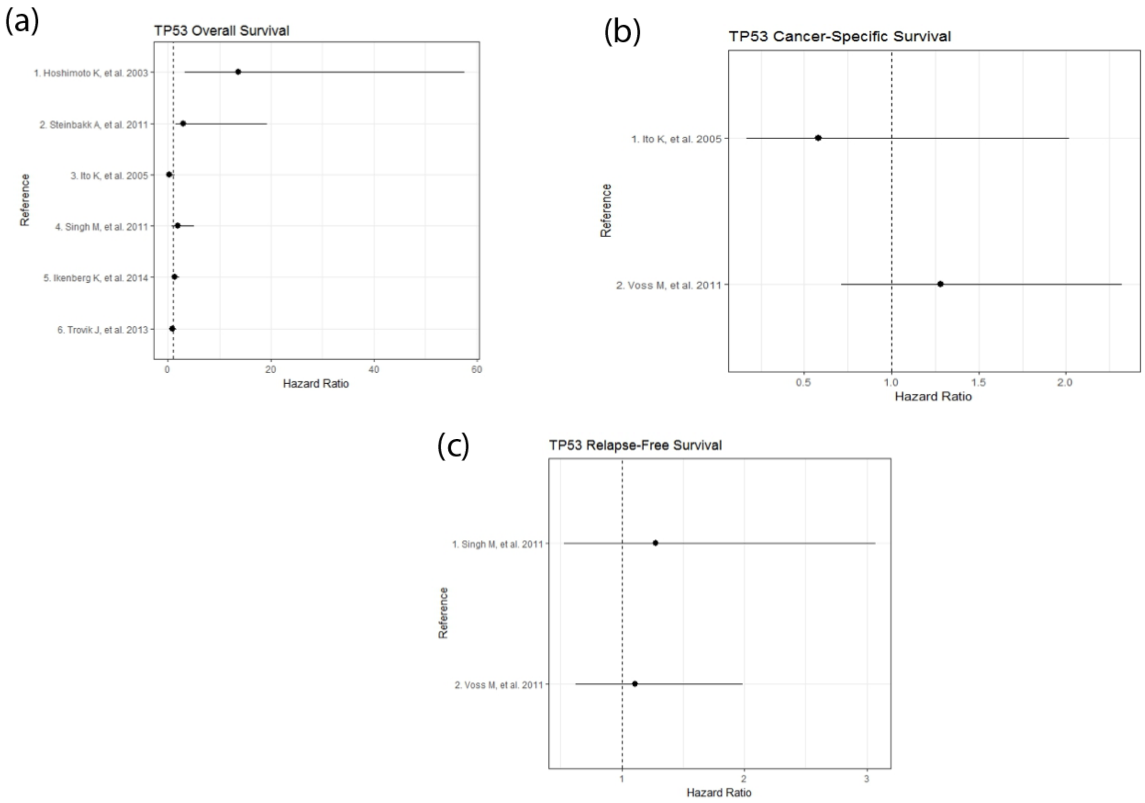

Supplementary Figure 2 Forest plots for the Hazard Ratio of mutated TP53 for (a) Overall survival; (b) Cancer-specific survival and (c) Relapse-free survival.
